# Polypharmacy and antidepressant acceptability in comorbid depression and type 2 diabetes: a cohort study using UK primary care data

**DOI:** 10.3399/BJGP.2022.0295

**Published:** 2023-01

**Authors:** Annie Jeffery, Cini Bhanu, Kate Walters, Ian CK Wong, David Osborn, Joseph F Hayes

**Affiliations:** Epidemiology and Applied Clinical Research Department, Division of Psychiatry, University College London, London, UK.; Epidemiology and Applied Clinical Research Department, Division of Psychiatry, University College London, London, UK.; Department of Primary Care and Population Health, Institute of Epidemiology and Health, University College London, London, UK.; Research Department of Practice and Policy, School of Pharmacy, University College London, UK; Centre for Safe Medication Practice and Research, Department of Pharmacology and Pharmacy, Li Ka Shing Faculty of Medicine, University of Hong Kong, Hong Kong.; Epidemiology and Applied Clinical Research Department, Division of Psychiatry, University College London, London, UK; Epidemiology and Applied Clinical Research Department, Division of Psychiatry, University College London, London, UK.

**Keywords:** antidepressants, depression, multimorbidity, polypharmacy, primary care, type 2 diabetes

## Abstract

**Background:**

Polypharmacy may increase the risk of drug interactions, side effects, and poor adherence; however, the impact of polypharmacy on antidepressant acceptability in individuals with type 2 diabetes (T2DM) is unknown.

**Aim:**

To investigate the association between number of prescribed medications and early antidepressant discontinuation in adults with T2DM.

**Design and setting:**

Cohort study using UK primary care data from the Clinical Practice Research Datalink between 1 January 2000 and 31 December 2018.

**Method:**

Cox regression with penalised B-splines was used to describe the association between the number of concurrently prescribed medications at the time of starting antidepressant treatment and each of the outcomes.

**Results:**

A total of 73 808 individuals with comorbid depression and T2DM starting antidepressant treatment for the first time were identified. A median of 7 concurrent medications were prescribed. Within 32 weeks, 44.26% (*n* = 32 665) of participants discontinued antidepressant treatment altogether, and 11.75% (*n* = 8672) of participants switched antidepressant agents. An inverse relationship between the number of concurrent medications and discontinuing antidepressant treatment altogether was found. The median of 7 concurrent medications was associated with a 65.06% decrease in early antidepressant discontinuation; hazard ratio 0.45, 95% confidence interval = 0.37 to 0.55. No evidence of an association between the number of concurrent medications and switching antidepressant agents was found.

**Conclusion:**

Early discontinuation of antidepressants is common in adults with T2DM; however, individuals with higher levels of concurrent polypharmacy may be more adherent to treatment. These are likely to represent individuals with worse physical or mental health. Individuals with lower levels of concurrent polypharmacy may benefit from adherence support.

## INTRODUCTION

Depression and type 2 diabetes (T2DM) are both major contributors to the global burden of disease,^1^ with a bidirectional relationship between depression and T2DM.^2^^,^^3^ The successful treatment of both conditions is important for the management of the other.

Antidepressants are recommended as a treatment option for individuals with moderate to severe depression and physical comorbidities;^4^ however, these recommendations are not condition specific and warn for clinicians to be aware of drug interactions. In order for antidepressants to be effective, National Institute for Health and Care Excellence (NICE) guidelines recommend a treatment duration of 6 months following the resolution of symptoms.^5^ However, in practice, antidepressant treatment is often discontinued early because of a lack of acceptability caused by ineffectiveness or intolerability.^6^^–^8

The use of multiple medications, or polypharmacy, is common in individuals with T2DM, with the need to control blood sugar levels, blood pressure, and cholesterol in most individuals, as well as the management of potentially numerous complications.^9^ However, polypharmacy may be associated with increased risk of drug interactions and side effects.^10^^,^^11^ Indeed, antidepressants specifically may cause side effects that exacerbate T2DM and/or its complications.^12^^,^^13^ Polypharmacy has also been shown to be associated with reduced medication adherence.^14^^,^^15^

Three systematic reviews found an improvement in depression symptoms following antidepressant treatment in people with T2DM; however, no evidence was found for the effect of polypharmacy on antidepressant acceptability.^16^^–^18

Early discontinuation of antidepressant treatment may be used to measure antidepressant acceptability in a real-world population, where, unlike many randomised controlled trials (RCTs), patients have comorbidities and polypharmacy.^19^ The authors of the present study aimed to describe, in individuals with comorbid depression and T2DM, the association between polypharmacy and:

- early discontinuation of antidepressant treatment (<32 weeks); and
- switching to an alternative antidepressant agent.

The authors hypothesised that for each additional concurrent medication prescribed, participants would be more likely to discontinue antidepressant treatment early or to switch antidepressant agents because of the increased risk of drug interactions, side effects, and reduced adherence.

**Table:** How this fits in

There is a bidirectional association between depression and type 2 diabetes (T2DM), therefore the treatment of each is important to the other. However, the use of multiple medications, or polypharmacy, may increase the risk of drug interactions, side effects, and poor adherence. Polypharmacy is common in individuals with T2DM, yet the impact of polypharmacy on antidepressant use in individuals with T2DM is unknown. This study revealed that individuals with higher levels of polypharmacy may be more adherent to antidepressant treatment, potentially owing to more severe depression and thereby an increased need for treatment.

## METHOD

A cohort study was carried out using data from the Clinical Practice Research Datalink (CPRD) GOLD and Aurum: a longitudinal dataset of pseudonymised electronic primary care records of >60 million people across 2000 primary care practices in the UK.^20^ The CPRD has been shown to be representative of the UK population with respect to age, sex, and ethnicity.^21^^,^^22^

The study period ran from 1 January 2000 to 31 December 2018.

### Inclusion criteria

Codelists used to identify records related to depression, T2DM, and antidepressants are provided in Supplementary Boxes S1 and S2. Individuals who met the study criteria for depression, T2DM, and first-time antidepressant use were included.

#### Criteria for depression

Individuals who had at least one record for depression symptoms (including, for example, low mood), diagnosis, or process of care. Those with records of mixed anxiety and depression were also included, but not those with records for anxiety without depression. Individuals who only had records for depression related to dementia, maternity, schizophrenia, or bipolar disorder were excluded as these are distinct disorders from depression.

#### Criteria for T2DM

Individuals who had at least two blood/serum glucose/HbA1C tests recorded above the threshold for T2DM plus one of the following recorded: diagnosis, symptom, or process of care record entries for T2DM; or antidiabetic medication prescriptions were included. In line with previous research that shows the necessity of cross validation for T2DM identification in electronic health records (EHRs),^23^ the authors excluded individuals who met any of the following criteria: possible type 1 diabetes mellitus (T1DM), identified <6 months between the date of the first recorded oral antidiabetic prescription and the first recorded insulin prescription; or gestational diabetes only, identified when record entries for T2DM and antidiabetic medication were only present during periods of pregnancy.

#### Criteria for first-time antidepressant use

The authors included individuals with comorbid depression and T2DM who received their first ever antidepressant prescription between the years 2000 to 2018, after their first diabetes-related record; where the first antidepressant prescribed was monotherapy with a common first-line antidepressant (citalopram, escitalopram, fluoxetine, mirtazapine, paroxetine, sertraline, or venlafaxine). Individuals with <6 months of antidepressant-free data before the date of their first antidepressant prescription were excluded to ensure that incident prescribing was identified.

### Outcomes

The researchers defined early discontinuation of the first antidepressant prescribed as any treatment duration lasting <7 months. This is in line with NICE recommendations, which state that antidepressant treatment should be maintained for 6 months after the remission of an episode of depression, allowing for 4–6 weeks for remission to be achieved.^5^

The primary outcome of this study was the discontinuation of antidepressant treatment altogether: defined as the absence of a recorded prescription for any other antidepressant within the first 60 days after the date of the last antidepressant prescription. The authors specified a gap of 60 days to account for a 1-month prescription (the median duration of antidepressant prescription), plus a maximum of 1 month to issue the subsequent prescription, in line with other research.^24^^,^^25^ This gap was considered a sufficient length of time for new antidepressant users who are unlikely to have stockpiled more medication.

The secondary outcome was switching antidepressant agents, defined as having a first recorded prescription for any other antidepressant within the first 60 days after the date of the last antidepressant prescription before the original antidepressant agent was discontinued.

Participants were censored if the treatment duration for the first course of antidepressant prescriptions reached >32 weeks, at the date of death, the date that their primary care practice registration ended, or 31 December 2018, whichever was sooner.

### Exposure

The main exposure was the number of different medications with at least one prescription recorded at the time of the first antidepressant prescription (index date) or up to 90 days prior. This did not include the antidepressant itself, but could include antidiabetic medications. The specification of 90 days was to allow for prescriptions of longer durations, which may be relevant for chronic conditions. Only pharmaceutical medications were included; topical medications, supplements, and vaccinations were excluded.

### Control

Participants who manage their diabetes through diet and exercise may not receive any concurrent medications. Thus, the reference category (control group) exposure value was zero concurrent medications (not including the antidepressant).

### Sensitivity analysis

A sensitivity analysis was performed, redefining the exposure variable (the number of concurrent medications) to distinguish ongoing polypharmacy (repeat prescriptions) from one-off prescriptions. To do so, at least two prescriptions within 180 days before the index date were required, with at least one of these being within 90 days. The reference category was individuals who did not have any repeat prescriptions meeting this criterion.

### Covariates

Calendar year, age, sex, ethnicity, and primary care practice were included as potential confounders. The inclusion of primary care practice enabled control for practice-level socioeconomic factors. Where ethnicity was missing, ‘White’ was recorded as it has been found in previous studies that the majority of individuals in UK EHRs with missing ethnicity are of White ethnicity.^26^ Comorbidities or diabetes stages were not included as covariates in the study because of collinearity with the main exposure.

### Statistical analyses

This study estimated the association between the number of concurrent medications and each outcome in univariable analyses then multivariable analyses, adding the aforementioned covariates. The primary care practice was included as a strata term to account for the clustering effect of each primary care practice.

Cox regression was used to investigate the association between the main exposure (number of concurrent medications) and each of the outcomes. Given the non-linear relationship between the number of concurrent medications and antidepressant discontinuation, it was necessary to transform the main exposure variable (number of concurrent prescriptions) using a penalised B-splines term. Spline functions enable the use of the linear Cox proportional hazards model where linear assumptions are not met, by fitting a number of linear functions to a non-linear relationship to provide interval estimates. While user-selected splines can introduce bias, the researchers used a penalised method to fit the splines, which balances flexibility against overfitting.^27^ The penalised fit defines the range of each spline, at which the interval estimates are made.

All above analyses were repeated for sensitivity with the redefined exposure variable for repeat prescriptions.

## RESULTS

A total of 73 808 participants with comorbid depression and T2DM, and who had started antidepressant treatment for the first time during the study period, were identified. The participant group was 52.03% female (*n* = 38 402) and had a median age of 63 years (interquartile range [IQR] 52–75). In terms of diabetes severity, 12.27% (*n* = 9057) were at an early stage where they received no pharmacological treatment and 14.41% (*n* = 10 635) were at the most severe stage requiring insulin therapy. The median number of concurrent prescriptions at the time of starting antidepressant treatment (including antidiabetic medication but excluding the antidepressant itself) was 7 (IQR 4–10). Full participant characteristics are shown in Table 1. The median treatment duration for the first antidepressant prescribed was 4.57 months (IQR 0.92–19.22). Early discontinuation of antidepressant treatment altogether was seen in 44.26% (*n* = 32 665) of participants, and switching from the first antidepressant to an alternative antidepressant agent was seen in 11.75% (*n* = 8672) of participants.

**Table 1.** Participant characteristics and descriptive analysis

**Characteristics**	**All participants (*N* = 73 808)**
**Age, years, median (IQR)**	63 (52–75)

**Sex, female, *n* (%)**	38 402 (52.03)

**Ethnicity group, *n* (%)**	
Asian	4413 (5.98)
Black	1805 (2.45)
White	41 607 (56.37)
Missing (imputed as White)	25 098 (34.0)
Mixed	366 (0.50)
Other	519 (0.70)

**Diabetes treatment stage, *n* (%)**	
Early stage (no pharmacological treatment)	9057 (12.27)
Metformin only	21 088 (28.57)
Second-line oral antidiabetics	33 028 (44.75)
Insulin	10 635 (14.41)

**Concurrent prescriptions at the time of starting antidepressant treatment, median (IQR)**	
Number of concurrent prescriptions^a^	7 (4–10)
Number of concurrent repeat prescriptions^a^	5 (3–8)

**Antidepressant prescribing outcomes**	
Duration of first antidepressant course, months, median (IQR)	4.57 (0.92–19.22)
Early discontinuation, *n* ( %)	32 665 (44.26)
Switched antidepressant agent, *n* (%)	8672 (11.75)
Censored <224 days (32 weeks), *n* (%)	3854 (5.22)

a Concurrent prescriptions were measured at/before the first recorded antidepressant medication; these include antidiabetic medications but exclude the antidepressant itself. IQR = interquartile range.

**Figure 1.**
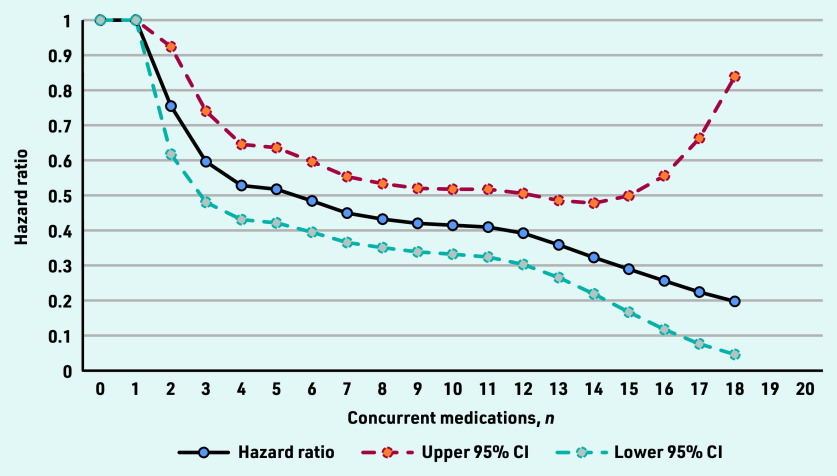
*Hazard ratios for the changing rate of discontinuing antidepressant treatment altogether, by the number of concurrent medications prescribed at the start of antidepressant treatment (adjusted for covariates).*

The authors found an inverse relationship between the number of concurrent medications prescribed at the time of starting antidepressant treatment and the rate of discontinuing antidepressant treatment altogether (Figure 1). The association began at two concurrent medications, with the rate of discontinuing antidepressant treatment altogether decreasing for each additional medication prescribed until 18 concurrent medications, at which point the maximum association was reached. In the fully adjusted model, the median of 7 concurrent medications was associated with a 65.06% decrease in the rate of discontinuing antidepressant treatment altogether (hazard ratio [HR] 0.45; 95% confidence interval [CI] = 0.37 to 0.5, Figure 1). Adjustment for covariates had a minimal effect on the model.

The inverse relationship remained in the sensitivity analysis, which redefined the exposure variable to include repeat prescriptions only. However, the size of the association was reduced, with seven concurrent repeat prescriptions being associated with a 38% decrease in the rate of discontinuation (HR 0.62; 95% CI = 0.57 to 0.67).

No statistically significant evidence was found of an association between the number of co-prescriptions prescribed at the start of antidepressant treatment and the rate of switching antidepressant agents.

The hazard ratio point estimates for all analyses and the number of people with each concurrent medication count are reported in Supplementary Tables S1–S5.

## DISCUSSION

### Summary

To the authors’ knowledge, this is the first study to investigate the impact of polypharmacy on antidepressant acceptability in adults with comorbid T2DM. This study found that the majority of participants (56%) either discontinued or switched their first antidepressant before the recommended duration.

The authors had hypothesised that polypharmacy would be associated with higher rates of antidepressant treatment discontinuation altogether; however, they found that participants who were prescribed more concurrent medications at the time of starting antidepressant treatment were less likely to discontinue antidepressant treatment. The authors had also hypothesised that polypharmacy would be associated with higher rates of switching antidepressant agents; however, no statistically significant evidence of an association between the number of concurrent medications and switching antidepressant agents was found.

The reasons for medication discontinuation are not captured within CPRD EHRs; however, these reasons may include ineffectiveness, intolerability, and non-adherence. The fact that an association between concurrent medication use and discontinuing antidepressants was seen, but not with switching antidepressant agents, may suggest that individuals with higher levels of polypharmacy are more adherent to medication overall and, therefore, less likely to discontinue antidepressant treatment early.

In the sensitivity analysis that redefined exposure to include only repeat prescriptions, a smaller inverse association with discontinuing antidepressant treatment altogether was seen. This may suggest that experiencing acute health events, or new or worsened health issues at the start of antidepressant treatment may additionally be associated with improved antidepressant adherence. Again, this may indicate more severe depression.

### Strengths and limitations

Routinely collected EHRs provide the opportunity to observe real-world antidepressant use in a complex population with comorbidities and concurrent polypharmacy. RCTs often exclude such individuals, and therefore may not be representative of the population of interest or be generalisable.^28^ In real-world clinical decision making, the commencement of antidepressant treatment and duration of use is based to a large extent on patient and/ or clinician preferences and behaviours. These are not possible to capture in RCTs, which use predefined interventions and endpoints.^28^

With a sample size of 73 808 participants, the present study is over 800 times larger than the largest RCT investigating antidepressant treatment outcomes in individuals with T2DM.^16^ This has enabled the authors to more precisely model the non- linear relationship between polypharmacy and antidepressant outcomes using penalised B-spline functions. An alternative option would be to categorise the number of medications into clinically relevant groups, for example, 1–4, 5–10, and so on. However, the present results show that the rate of antidepressant discontinuation altogether decreases for each additional concurrent medication — a clinically relevant finding that would have been lost with wider categorisations.

As the minimum number of concurrent medications prescribed at the time of starting antidepressant treatment was 0, and a sufficient number of participants met this criterion, this was selected as the baseline value for which spline point comparisons were made. Participants who were prescribed 0 concurrent medications at the start of antidepressant treatment would either be those at the earliest stages of T2DM, or those who were very non-adherent. While participants taking ≥1 medication may be more representative of individuals with T2DM, the present study’s model did not fit a spline point at the value of 1 concurrent medication, showing that there was no evidence of a difference between 1 and 0 concurrent medications. Thus, the comparisons were made with individuals with no polypharmacy.

With the exception of private prescriptions, all prescribing outside a secondary care setting is recorded in primary care. Prescribing is electronic, and it is not possible to issue a prescription without it being entered accurately on a patient’s EHR; therefore, the authors have confidence in the completeness and accuracy of their outcome and exposure variables. However, as the rationale behind prescribing decisions is not captured by EHR data, the full interpretation of the present results can only be speculative. Qualitative research is required to explore the reasons why individuals with comorbid depression and T2DM are less likely to discontinue antidepressant treatment early when they are prescribed more concurrent medications.

This study was unable to account for depression severity as no suitable variables were available in the dataset. The study inclusion criteria for depression had depression at any level of severity; therefore, the associations that were found with polypharmacy may instead be markers of depression severity, which may increase the requirement to continue antidepressant treatment for the full course of treatment.

An additional limitation of EHR research is that data concerning the wider determinants of health and behaviours, which may influence these findings, are not available.

### Comparison with existing literature

The median duration of treatment (4.57 months) is lower than in the UK general population, where the average duration has been reported to be 6 months,^29^ suggesting that antidepressant acceptability is lower for individuals with comorbid T2DM.

Several studies investigating adherence to somatic medication in individuals with T2DM have also found a positive association between polypharmacy and adherence.^30^^–^32 This may have been because of increased contact with healthcare services.^29^ Also, in the general population, distrust of clinicians has been reported as a reason for early antidepressant discontinuation^33^ — this could be relevant for individuals with lower levels of polypharmacy. Alternatively, previous research has found associations between higher levels of polypharmacy and increased depression symptoms.^34^ Therefore, individuals with higher levels of polypharmacy may be in a state of more severe depression, whereby antidepressant treatment cannot be interrupted. Similarly, individuals with lower levels of polypharmacy may discontinue antidepressant treatment early because they feel better and no longer feel they need antidepressants.

### Implications for practice and research

Early discontinuation of antidepressant treatment in adults with comorbid T2DM is common and may jeopardise the treatment of depression; however, there was no evidence of a negative effect from polypharmacy on antidepressant acceptability in the present study group. Conversely, individuals with comorbid depression and T2DM receiving higher numbers of concurrent medications may be more adherent to antidepressant treatment. These individuals could be more adherent to medications overall, or they could be more severely depressed requiring the completion of the recommended duration of antidepressant treatment. Individuals with fewer concurrent medications — who may represent those with less complex T2DM, fewer comorbidities, less contact with services, or who are less adherent with treatment — are potentially at increased risk for early antidepressant discontinuation. Depression may be undertreated in these individuals, which could lead to worse diabetic health outcomes. Increased monitoring and adherence support may benefit such individuals.

Further research is required to differentiate the potential reasons for early discontinuation, the impact of depression severity and wider determinants of health, and to understand the safety of antidepressant use alongside commonly prescribed medications in T2DM.

## Data

Data are available from the CPRD following study-specific protocol approval via CPRD’s Research Data Governance Process.
